# Effects of Picoxystrobin and 4-n-Nonylphenol on Soil Microbial Community Structure and Respiration Activity

**DOI:** 10.1371/journal.pone.0066989

**Published:** 2013-06-20

**Authors:** Marianne Stenrød, Sonja S. Klemsdal, Hans Ragnar Norli, Ole Martin Eklo

**Affiliations:** Norwegian Institute for Agricultural and Environmental Research (Bioforsk), Ås, Norway; Wageningen University, The Netherlands

## Abstract

There is widespread use of chemical amendments to meet the demands for increased productivity in agriculture. Potentially toxic compounds, single or in mixtures, are added to the soil medium on a regular basis, while the ecotoxicological risk assessment procedures mainly follow a chemical by chemical approach. Picoxystrobin is a fungicide that has caused concern due to studies showing potentially detrimental effects to soil fauna (earthworms), while negative effects on soil microbial activities (nitrification, respiration) are shown to be transient. Potential mixture situations with nonylphenol, a chemical frequently occurring as a contaminant in sewage sludge used for land application, infer a need to explore whether these chemicals in mixture could alter the potential effects of picoxystrobin on the soil microflora. The main objective of this study was to assess the effects of picoxystrobin and nonylphenol, as single chemicals and mixtures, on soil microbial community structure and respiration activity in an agricultural sandy loam. Effects of the chemicals were assessed through measurements of soil microbial respiration activity and soil bacterial and fungal community structure fingerprints, together with a degradation study of the chemicals, through a 70 d incubation period. Picoxystrobin caused a decrease in the respiration activity, while 4-n-nonylphenol caused an increase in respiration activity concurring with a rapid degradation of the substance. Community structure fingerprints were also affected, but these results could not be directly interpreted in terms of positive or negative effects, and were indicated to be transient. Treatment with the chemicals in mixture caused less evident changes and indicated antagonistic effects between the chemicals in soil. In conclusion, the results imply that the application of the fungicide picoxystrobin and nonylphenol from sewage sludge application to agricultural soil in environmentally relevant concentrations, as single chemicals or in mixture, will not cause irreversible effects on soil microbial respiration and community structure.

## Introduction

Various practices have been promoted to meet the demands for increased productivity of agricultural areas, including the use of mineral fertilizers and pesticides as well as the use of organic amendments. The potential risk of non-target effects of pesticides in soils is evident, and has been widely studied. Specific attention is now given to the use of organic amendments originating from sewage sludge. Sludge has been shown to contain high levels of many organic chemicals (e.g. nonylphenols, PAHs a.o.) that might exert ecotoxicological effects upon soil addition, e.g., [1], [2]. Potentially toxic compounds, single or in mixtures, are added to the soil medium on a regular basis, while the ecotoxicological risk assessment procedures mainly follow a chemical by chemical approach focusing on establishing dose-response relationships for soil fauna. The use of sewage sludge on agricultural fields where pesticides are sprayed regularly as part of conventional farming practices calls for increased attention to the potential combined effects of pesticides and known contaminants in sewage sludge.

Sewage sludge, potentially used for application on agricultural soil, is known to contain considerable amounts of nonylphenol [2]–[4] – an industrial by-product and degradation product of nonylphenol ethoxilate plasticizers. The occurrence, fate and toxicity of nonylphenol in the environment have been reviewed by Soares and co-workers [4], pointing at knowledge gaps as well as identified challenges with nonylphenol in soil including occurrence and possible accumulation in soil following sludge application, reduced degradation rates in soil due to sorption and reduced bioavailability, and potential toxic effects on soil microorganisms. Nonylphenol is also included on the list of priority substances of the Water Framework Directive [5] and, hence, require further attention to clarify its potential effects in soil. Picoxystrobin is a strobilurin fungicide [6] for spraying in cereals, with a maximum of one spraying per season in Norway. Picoxystrobin inhibits mitochondrial respiration by blocking electron transfer at the Qo centre of cytochrome bc1 [7]. Picoxystrobin is reported to have a high acute toxicity to earthworms (LC_50_ at 6.7 mg kg^−1^) [8], and field assays of earthworm toxicity indicate acute toxic effects even at recommended doses for use [9], which might be caused by heavy rain shortly after spraying forcing the earthworm to migrate to the surface. Potentially negative effects on soil microbial nitrogen and carbon mineralization activity are shown to be transient (dose: 750 g ha^−1^, duration: 28 days) [8]. Picoxystrobin require continued attention due to its demonstrated potentially negative effects to earthworms.

The importance of soil microbes and their activity in the functioning of soils, e.g., [10], [11] justify their thorough investigation in risk assessments [12]–[14]. The development of genomic techniques over the last decades has made detailed studies of the soil microbial community possible, beyond the scope of broad-scale measures like substrate induced respiration. DNA extraction from soil followed by different molecular approaches to determine the genetic diversity and quantify the presence of single organisms or groups of related organisms in a soil sample, have been employed successfully in studies of species and functional diversity in agricultural soils [15]–[17]. According to OECD guidelines for the testing of chemicals carbon [18] and nitrogen [19] transformation tests (with cut-off criteria of 25% effect) are the recommended methods to assess effects concentrations of chemicals on the soil microbial community. In research, soil respiration is commonly used to assess effects of pesticides and other chemicals on soil microbes. These are measures linked to the activity level of the soil microbial community, but are crude measures that do not necessarily reveal all relevant effects. The low percentage of soil microorganisms we are able to culture ex situ stresses the need for employing molecular and genomic methods suitable for terrestrial ecotoxicological studies. T-RFLP-analysis is one much used technique showing good results when looking at effects of different environmental conditions and chemical stressors on soil microbial communities, e.g., [16], [17], [20]. But there are many important methodological aspects to be considered when interpreting the results, e.g., [21], [22], including choice of primers and restriction enzymes, procedure for noise reduction and profile alignment, and statistical analysis using relative abundance or presence/absence data.

The main objective of this study was to assess the effects of picoxystrobin and nonylphenol, as single chemicals and mixtures, on soil microbial community structure and respiration activity in an agricultural sandy loam soil. The choice of compounds is based on their individual occurrence and effects in the environment, and expected potential for co-occurrence in the field, although the latter is scarcely documented. Further, they were chosen to represent an example of combined effects of agricultural and industrial contaminants with their expected independent effect mechanisms due to differences in mode of action. Effects of the chemicals were assessed through measurements of soil microbial respiration activity and soil bacterial and fungal community structure fingerprints, together with a degradation study of the chemicals, through a 70 d incubation period. The different measures showed corresponding results in support of a conclusion that a mixture situation with picoxystrobin and nonylphenol in soil will not increase the potential negative effects of picoxystrobin on the soil microbial community.

## Materials and Methods

### Soil

Bulk soil sampling in the top 10 cm of the plough-layer of an agricultural field at Norderås, Ås, South East Norway (59°41′14′′ N, 10°46′22′′ E), was done in middle of August 2008. After sieving (4 mm mesh) the soil was physically and chemically characterized (Table 1; all analyses performed in accordance with recognized laboratory standards at Analycen AS/Eurofins Norway) and stored moist at 4°C for about 3 weeks before use in laboratory experiments. No specific permits were required for the described field soil sampling and subsequent lab studies.

**Table 1:** Selected physical and chemical characteristics of the studied soil.

Parameter	Value	Unit
pH	6.3	
TOC – Organic C	1.1	%
Kjeldahl N	0.0986	%
C/N	11	
Tot C	2.0	%
Tot N	0.07	%
CEC	11.2	meq/100 g
Coarse (>2 mm)	13.7	%
Sand (2.000-0.060)	68.3	%
Silt (0.060-0.002)	20.7	%
Clay (<0.002)	11.0	%

Analysis performed by Analycen AS/Eurofins Norway.

The Water Holding Capacity (WHC) was estimated on sieved soil gently packed in plastic columns. After wetting with Milli-Q-water overnight, excess water was allowed to drip off during 1 h the next day, and the gravimetric moisture content was measured after drying overnight at 105°C.

### Chemicals

Treatment solutions were prepared from analytical grade picoxystrobin (Riedel-de Häen) and 4-n-nonylphenol (Fluka) purchased from Sigma-Aldrich. The chemicals were dissolved in acetone (4 ml portions; Lab-Scan) and added to quartz sand (27 g portions; Sigma-Aldrich). After evaporation of the acetone (about 30 minutes) the quartz sand with chemical was mixed into batches of moist soil (2.7 kg dry weight equivalent (dw eq.), 60% WHC) giving 2 or 10 mg picoxystrobin kg^−1^ soil (dw eq.), or 0.5 or 10 mg 4-n-nonylphenol kg^−1^ soil (dw eq.) as single treatments as well as all mixture combinations (Table 2). A solvent control (acetone added to quartz sand, left to evaporate before addition to soil) and an untreated control (soil with no addition of chemical) were included in the experiment.

**Table 2:** Overview of picoxystrobin and 4-n-nonylphenol treatments in the incubation experiment.

Treatment	Picoxystrobin (PI)		4-n-nonylphenol (NP)	
	2 mg kg^−1^ dry soil	10 mg kg^−1^ dry soil	0.5 mg kg^−1^ dry soil	10 mg kg^−1^ dry soil
PI low	**x**			
PI high		**x**		
NP low			**x**	
NP high				**x**
Mix low	**x**		**x**	
Mix NP high	**x**			**x**
Mix PI high		**x**	**x**	
Mix high		**x**		**x**
Control				

The lowest test concentration for picoxystrobin was set from reported levels for transient effects on carbon and nitrogen mineralization (750 g ha^−1^) [8], considering that picoxystrobin has been shown to sorb to the top cm of soil (50–70% interception, sorption in top 1 cm of soil), although recommended doses in Norway are below these levels. The worst case concentration was set to 10 mg kg^−1^ surpassing reported acute toxicity levels for earthworm (LC_50_ = 6.7 mg kg^−1^) [8]. The lowest test concentration for nonylphenol was set to the Predicted Initial Environmental Concentration (PIEC) estimated through a risk assessment made by the Norwegian Scientific Committee on Food Safety [2]; application of sludge containing mean expected amounts of nonylphenol (32 mg kg^−1^) at rates in accordance with Norwegian regulations (40 tonnes ha^−1^). From this a worst case concentration was set to 10 mg kg^−1^ considering a risk of reduced incorporation depth and possible initial effects.

### Experimental Set-up

An incubation experiment was set up with all chemical treatments in five replications and the control treatments in four replications. Subsamples of about 100 g dw eq. of sieved, moist soil (60% WHC) in plastic (polypropylene) containers were placed in air-tight glass jars together with small beakers with NaOH-solution (10 mL, 1 M) to trap CO_2_ evolved from the soil. All treatments were set up with five repeats to enable destructive sampling of five and four replicates, of chemical treatments and the control respectively, on each sampling occasion through the 70 d incubation period. A total of 244 samples were incubated at 20°C in the dark. Samples were taken on six time-points; before addition of chemicals (4 samples), and after 1, 7, 14, 28 and 70 days of incubation (48 samples on each occasion). Each sample was homogenized and subsamples taken for chemical residue analysis (25 g), and DNA extraction (30 g). In addition, the general microbial activity level in the soil was monitored by weekly replacement and analysis of the NaOH-traps, giving a total of 11 time-points with measurements.

### Total Soil Microbial Activity

The total activity of soil microbial biomass was followed by respirometric measurements [23]. The CO_2_ in the NaOH traps was determined by colourimetric continuous flow analysis (AutoAnalyzer3, Bran+Luebbe, Germany). Results are reported as mg CO_2_-C kg^−1^ dry soil.

### Soil Microbial Community Structure

#### DNA extraction procedure

Soil samples were homogenized (30 g moist soil +30 mL Milli-Q-water) in a mill (3 min forward +3 min reverse spin; Retsch PM 400) before extraction of DNA according to the Fast DNA Spin Kit for Soil (QBiogene) manual. In short, soil suspension (400 µL) was added to Lysing Matrix E Tubes before homogenization with the FastPrep® instrument (30 sec at speed 5.5; QBiogene, MP Biomedicals) and centrifugation (14000×g, 3 min). DNA was purified with guanidine thiocyanate (5.5 M, Sigma) twice (1 mL and 600 µL) after DNA binding to the Binding Matrix Suspension, before elution (100 µL sterile distilled water) from Spin^TM^Filters. The eluate was further purified through Micro Bio-Spin Chromatography columns (Bio-Rad Laboratories Ltd.) packed with polyvinylpolypyrrolidone (PVPP) (Sigma) (4000×g, 5 min), and stored at −80°C.

#### PCR procedure

PCR reactions were run with fluorescently labelled PCR primer sets (Applied Biosystems), both forward and reverse primer labelled. After testing of 8 primer sets, chosen from previously published T-RFLP studies, PCR reactions were run for all samples with three selected primer sets and fluorescence labelling (Table 3).

**Table 3:** Primer sets used in the experiments.

Primerset no.	Primer	Sequence	Reference
1	63F^1^	CAG GCC TAA CAC ATG CAA GTC	[24]
	1087R^2^	CTC GTT GCG GGA CTT ACC CC	[24]
2	EF4^1^	GGA AGG GRT GTA TTT ATT AG	[25]
	Fung5^2^	GTA AAA GTC CTG GTT CCC C	[25]
3	ITS1F^3^	CTT GGT CAT TTA GAG GAA GTA A	[26]
	ITS4^4^	TCC TCC GCT TAT TGA TAT GC	[27]

Fluorescence labeling: ^1^6FAM,

2 VIC,

3 PET,

4 NED.

All PCR reactions were run with 1 µL template and 1 µL of forward and reverse primer (10 pmol/µL) in TaqMan® Environmental Master Mix 2.0 (12.5 µL; Applied Biosystems) made up to a total reaction volume of 25 µL with sterile distilled water, on GeneAmp® PCR system 9700 (Applied Biosystems) or PTC-200 (MJ Research) thermal cyclers (40 cycles). An annealing temperature of 55°C was used for all primer combinations. PCR-products were purified with GenElute^TM^PCR clean-up kit (Sigma-Aldrich) according to the instructions from the supplier, before restriction digestion and T-RFLP.

#### T-RFLP procedure

After initial testing of a range of restriction enzymes (AluI, DdeI, HaeIII, HinfI, MboI, MspI, RsaI, TaqI, HhaI, MvnI, BstUI) on random samples, MspI and HaeIII, and MspI and HinfI were chosen for restriction of PCR products from bacteria and fungi, respectively. Restriction with the different enzymes were run in separate reactions and analysed separately. Analysis was performed on an ABI 3730 DNA Analyzer (Applied Biosystems, Foster City, USA) after mixing of 1 µl sample (template) with 8.75 µl Hi-Di formamid™ (Applied Biosystems) and 0.25 µl GeneScan™ 500 LIZ size standard (Applied Biosystems). The results reported for the T-RFLP analyses are based on relative comparisons of fingerprints, to show relative effects of different chemical treatments and comparison with a soil sample not treated with chemicals.

### Degradation Study

Residues of picoxystrobin and 4-n-nonylphenol were extracted from 5 g subsamples of soil after mixing with 1.0 g of dehydrated MgSO_4_ (purum, Fluka, Sigma-Aldrich GmbH) and 10 mL acetonitrile (Pestiscan, LAB-SCAN POCH SA, Gliwice, Poland) in 50 ml centrifugal tubes. One µg of 4-n-nonylphenol-2,3,5,6-d4-OD (99.4%, Chiron, Trondheim, Norway) was added as internal standard. After a short homogenization (10–15 sec. whirl mix) the samples were extracted by end-over-end shaking (1 h; Reax2, Heidolph). Extraction efficiencies derived from analysis of sterilised (autoclaved) soil at 117±2.0% and 108±0.0% for 4-n-nonylphenol and picoxystrobin, respectively. After centrifugation (1800×g, 5 min), 1.5 ml of the supernatant was transferred to GC-vials for analysis on GC-MS. Calibration standards at 0.001, 0.01, 0.05, 0.2, 1.0 and 5 µg ml^−1^ where prepared by diluting stock solutions of picoxystrobin (98%, Dr. Ehrenstorfer, Augsburg, Germany) and 4-n-nonylphenol (99.2%, Chiron, Trondheim, Norway) with acetonitrile. To balance the matrix in the samples a GC-vial was added 1 ml of a blank soil extract which was evaporated to dryness. One ml of each calibration standard was added to the GC-vial together with 0.1 µg 4-n-nonylphenol-2,3,5,6-d4-OD as internal standard. The measurements were performed on an Agilent 6890 gas chromatograph connected to an Agilent 5973 mass spectrometer using ChemStation Software version D.03.00. The gas chromatograph was equipped with a Gerstel (Mühlheim Ruhr, Germany) programmable temperature vaporizing (PTV) injector with a sintered liner. The separation was performed using a fused silica column (HP-5MSI 30 m width, 0.25 mm internal diameter, 0.25 µm film thickness, J&W Scientific) connected to a 2.5 m methyl deactivated pre column (Varian Inc., Lake Forest CA, USA) of same internal diameter as the analytical column. The temperature program was as follows; 65°C held for 1.5 min, 20°C min^−1^ to 120°C, held for 0 min, 20°C min^−1^ to 300°C, held for 0.5 min, total runtime 13.75 min. The PTV program was as follows: solvent vent temperature 60°C held for 1.40 min with a vent flow at 200 ml min^−1^. After 1.42 min the split valve was closed and the injector temperature increased by 720°C min^−1^ to 250°C and kept for 2 min. Injection volume 10 µl. The mass spectrometer was operated in selected ion monitoring mode with target/qualifier ions as follows: 4-n-nonylphenol-D4: m/z = 111/224, 4-n-nonylphenol: m/z = 107/220 and picoxystrobin: m/z = 145/335. Transfer line temperature was set at 280°C, ion source temperature at 230°C and quadrupole temperature at 150°C.

Biological degradation of picoxystrobin and 4-n-nonylphenol was verified by spiking selected matrices (quartz sand, unsterile soil, sterilized soil) at 0.05 µg g^−1^ and left in the dark at room temperature (20±2°C) for 24 h before extraction and analysis as described above. The soil was sterilised by autoclaving (121°C, 20 min, x2) (Matachana S1000).

### Data Processing and Statistical Analysis

First-order rate constants for organic carbon mineralization were estimated by linear regression (SigmaPlot 11.0, SYSTAT) using mean cumulative values for CO_2_-C evolved, from the equation ln*S* = ln*S_0_*+ *kt*, where *S* is the amount of CO_2_-C evolved at time t (mg kg^−1^ dry soil), *S_0_* is the initial amount of mineralization product, *k* is the rate constant (d^−1^), and *t* is the time (d). Due to the non-normal distribution of our CO_2_-measurements, non-parametric analysis was utilized to test for differences between treatments; i.e. Kruskall Wallis test for equality of medians for all treatments, and Mann-Whitney test for equality of medians between selected treatments (Minitab 15, Minitab Inc.).

Handling of T-RFLP data with binning/identification of alleles, normalization (within project; i.e. for all samples independent of plate) and (automatic) alignment of profiles was done with GeneMapper 4.0 (Applied Biosystems) based on a 4 basepair (bp) bin width (assumed to give the more stable number of bins based on a screening of bin widths from 0.5 to 10 bp in 0.5 bp increments) and a threshold of 50 (default). Obvious pull-up peaks were removed from the dataset before further analysis. No further trimming of the data was done (i.e. no small peaks removed) to avoid exclusion of potentially important peaks for the microbial diversity analysis. Size (bp), area and height data were exported to an excel spreadsheet (Excel 2007, Microsoft corp.) for calculation of average size of each identified peak and calculation of relative peak height and area for all peaks within each individual sample. All peak data for each sample was assembled before statistical analysis, keeping the data for each primer set separate. The terminal fragments labelled from the forward primer as well as the fragments labelled from the reverse primer from each of the restriction reactions, were included in a single data row for each sample before analysis. The data were found to be not normally distributed (i.e. skewed due to rarely occurring peaks). Relative peak heights were analysed with non-metric multidimensional scaling (nmMDS) in R-software (v. 2.9.1) [28] using the metaMDS routine in the vegan package [29] with k = 2 or 3 dimensions, the isoMDS routine in the MASS package [30] with k = 2 dimensions, and with principal coordinates analyses using the PCO routine in R. All analyses resulted in similar patterns and the results presented are from metaMDS (k = 3 dimensions) as these gave the best fit to the observed data (STRESS below 0.10) [31], [32]. Results are reported as two and three-dimensional plots showing the clustering of treatments at the different time-points of analysis through the incubation period. The samples taken before addition of chemical and onset of the incubation period (original soil sample) were included in the analysis of all time-points as a reference. Further, analysis of similarity was performed with the ANOSIM routine in PAST-software [33] with Bray-Curtis distance measure utilized in all tests. Results are given as ANOSIM R of a value between 0 and 1, where values close to 1 indicate large differences between treatments.

The observed T-RFLP patterns for the untreated and solvent control were not statistically significant different as analysed by nmMDS, and the results presented focus on differences between chemical treatments and the solvent control. This was to avoid overestimating any effects of the studied chemicals. A similar clustering of T-RFLP fingerprints was observed for the results obtained from using primer sets 2 and 3 (i.e. soil fungal community fingerprints; Table 3). Hence, the presented results only include primer set 3.

Soil degradation half-lives (DT50) for picoxystrobin and 4-n-nonylphenol was estimated from results from the degradation study, and calculated according to first-order kinetics from the equation DT50 = log_10_2/b, where b is the regression coefficient estimated through a linear regression procedure. All measured values were utilized in the estimation of the DT50 values except for two extreme outliers being removed before statistical analysis of the data. Due to the five replicates for each treatment this could be done without compromising the results of the data analyses, as the analyses for the affected treatments were still based on four replicate samples. Due to some differences in the observed degradation pattern for the chemicals at low and high concentrations, the soil half-lives were estimated separately for these. The estimated half-lives are based on data from both the relevant single chemical and mixture treatment, as the observed degradation pattern did not differ significantly between these. As an example DT50 for 2 mg picoxystrobin kg^−1^ dry soil was estimated using data from both the single chemical treatment and the two mixture treatments with this concentration of picoxystrobin. The estimation was done assuming bi-phasic degradation of both chemicals, with both phases sufficiently described by first-order degradation kinetics and, hence, possible to estimate through linear regression.

A significance level of 5% was used for hypothesis testing.

## Results

### Total Soil Microbial Activity

**Figure 1:**
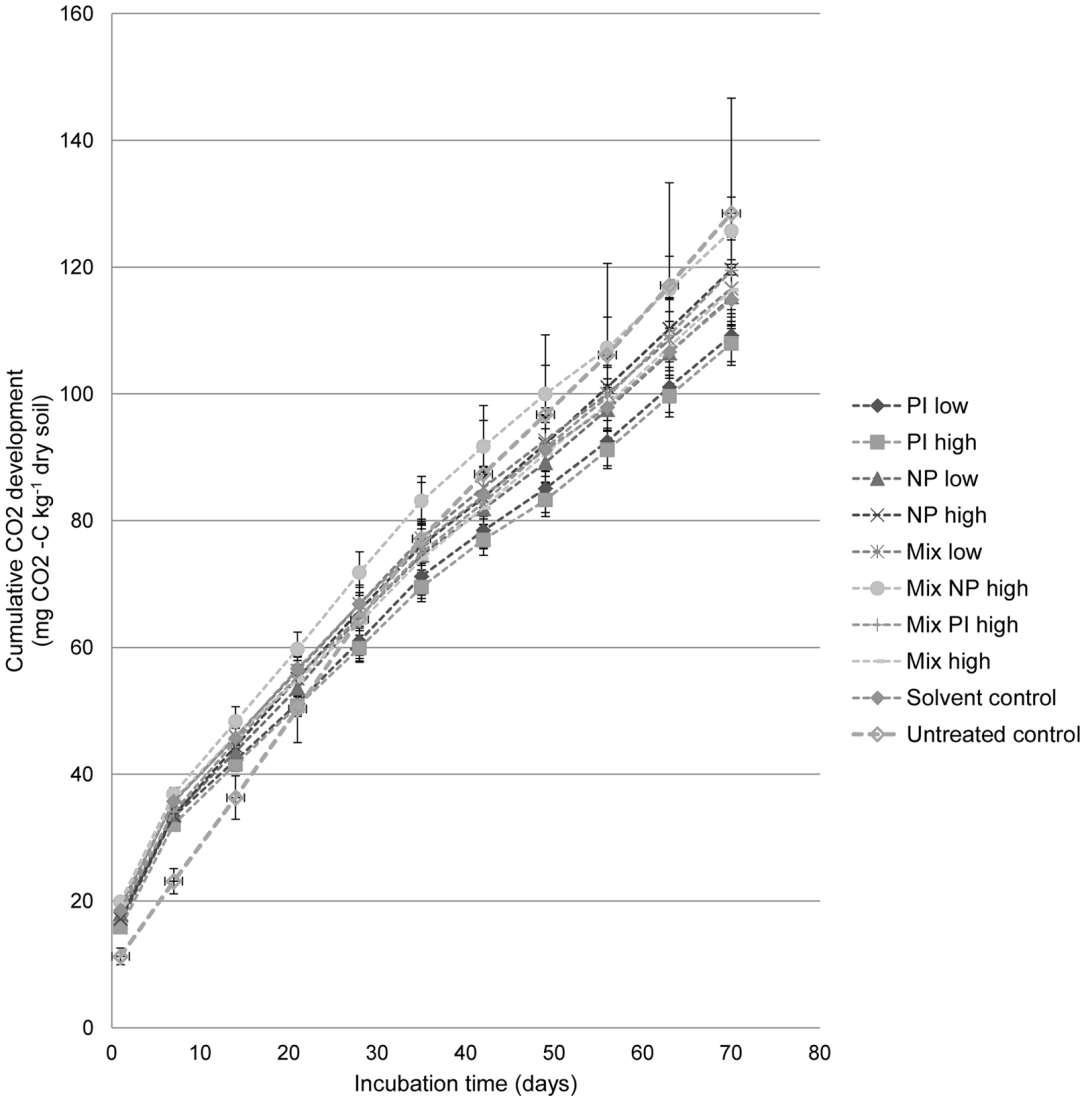
Effects of picoxystrobin and 4-n-nonylphenol on soil microbial respiration activity. Cumulative CO_2_ development in a sandy loam soil during a 70 d incubation period after treatment with picoxystrobin and 4-n-nonylphenol as single chemicals and in mixture. Mean values ± SD. Legends: Treatments with picoxystrobin (PI) or 4-n-nonylphenol (NP) or both chemicals (Mix), in low (2 mg kg^−1^ PI, 0.5 mg kg^−1^ NP) or high (10 mg kg^−1^) concentrations. Cf. Table 2 for full details.

No statistically significant differences between the treatments could be detected from observations of cumulative CO_2_-C development after 70 d and estimated first-order rate constants for organic C-mineralization (Table 4). However, cumulative respiration curves (Fig. 1) indicated a tendency of reduced respiration activity in treatments with picoxystrobin as a single chemical, and increased respiration activity in treatments with high concentrations of nonylphenol (single chemical and mixture). The untreated control showed a lower respiration rate in the beginning of the period and a higher respiration rate at the end of the period, as compared to the other treatments. This difference did not result in any statistically significant difference in cumulative CO_2_-C respiration, but indicated an effect of the solvent (acetone) used in the experiments.

**Table 4:** Organic C mineralization in soil.

Treatment	Organic C mineralization		
	Cumulative CO_2_-C	Rate constant	
	(mg CO_2_-C/kgdry soil 70d)	(k_totC_, 10^−4^ day^−1^)	r^2^
PI low	109.2±4.1^a^	1.7±0.17^b^	0.82
PI high	108.0±3.5	1.6±.0.17	0.83
NP low	115.3±4.6	1.7±0.18	0.83
NP high	119.6±5.1	1.8±0.17	0.85
Mix low	116.6±4.5	1.8±0.19	0.81
Mix NP high	125.7±5.3	1.9±0.20	0.83
Mix PI high	119.5±6.8	1.8±0.18	0.84
Mix high	116.4±7.9	1.8±0.19	0.81
Solvent control	114.9±3.9	1.8±0.19	0.80
Untreated control	128.5±18.2	1.7±0.19	0.96
Total		1.8±0.05	0.83

a SD (standard deviation of measurements),

b SE (standard error of estimate).

The indications from the latter were supported by analysis of the separate measurements of CO_2_ development from the soil samples at 11 time-points through the 70 d incubation period. Kruskall Wallis test for equality of medians between the treatments indicated statistically significant differences (p<0.001) when looking at all the individual CO_2_ measurements in the analysis separately (i.e. not cumulative values at 70 d). The individual ranking of the treatments (data not shown) gave the solvent control a rank close to the overall rank (i.e. mean), while treatments with picoxystrobin as a single chemical (2 and 10 mg kg^−1^ dry soil) were indicated to cause a reduction in respiration activity, through a rank statistically significant lower than the overall rank. High concentrations of 4-n-nonylphenol (10 mg kg^−1^ dry soil) were indicated to cause an increase in respiration activity, through a rank statistically significant higher than the overall rank. This was also the case for the untreated control.

These indications were further supported by results from Mann-Whitney tests for equality of treatment medians, comparing the treatments two by two (test criteria: p<0.05). High concentration of 4-n-nonylphenol (10 mg kg^−1^ dry soil) in mixture with low concentration of picoxystrobin (2 mg kg^−1^ dry soil) gave statistically significant higher CO_2_ development than treatments with picoxystrobin as a single chemical (2 and 10 mg kg^−1^ dry soil), low concentration of 4-n-nonylphenol (0.5 mg kg^−1^ dry soil), low concentration mixture (2 and 0.5 mg kg^−1^ dry soil of picoxystrobin and 4-n-nonylphenol, respectively) as well as the solvent control. Further, treatments with picoxystrobin as a single chemical (2 and 10 mg kg^−1^ dry soil) gave statistically significant reduction in CO_2_ development compared to all mixture treatments, high concentration of 4-n-nonylphenol (10 mg kg^−1^ dry soil) as a single chemical, and the untreated control.

### Soil Microbial Community Structure

**Figure 2:**
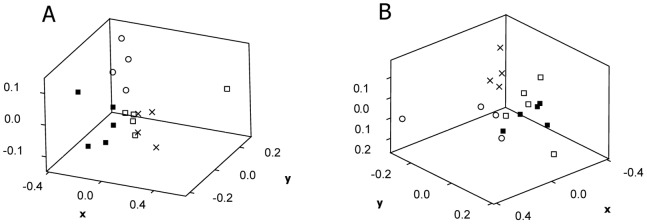
Effects of picoxystrobin on soil fungal community structure. Effects of picoxystrobin (PI) on soil fungal community structure, as shown from nmMDS of data from T-RFLP analyses after 7 (a) and 70 (b) days of incubation. Legends: Treatment PI low (□; 2 mg kg^−1^ dry soil) and PI high (▪; 10 mg kg^−1^ dry soil), solvent control (×; no chemical added) and original soil sample (○).

The fungicide picoxystrobin was found to have a statistically significant effect on the soil microbial community structure, as found from nmMDS and ANOSIM of relative peak height data from T-RFLP analyses (Table S1). Looking at the effects of single chemical treatments in bacteria (Fig. S1) the largest segregation between treatments was observed after 7 d, with an overall ANOSIM R of 0.65 (p = 0.0001). Here there was a statistically significant segregation between the low and high concentration treatment (R = 0.42; p = 0.018), indicating a concentration effect, and also a significant effect compared to the solvent control at low levels of picoxystrobin in the soil (R = 0.44, p = 0.04). Large variability was found in the data for the effects of picoxystrobin on the soil fungal community structure, with the largest spread in the data as shown by nmMDS after 7 d (Fig. 2a, Fig. S2) (R = 0.47, p = 0.0001). However, an effect due to different treatment concentrations of picoxystrobin was evident until 28 d (R = 0.38, p = 0.017) (Fig. S2). There was still a statistically significant difference between the single chemical treatments and the solvent control at 70 d for both bacteria (Fig. S1) and fungi (Fig. 2b), with an overall ANOSIM R of 0.65 and 0.4 (p = 0.0001), respectively.

**Figure 3:**
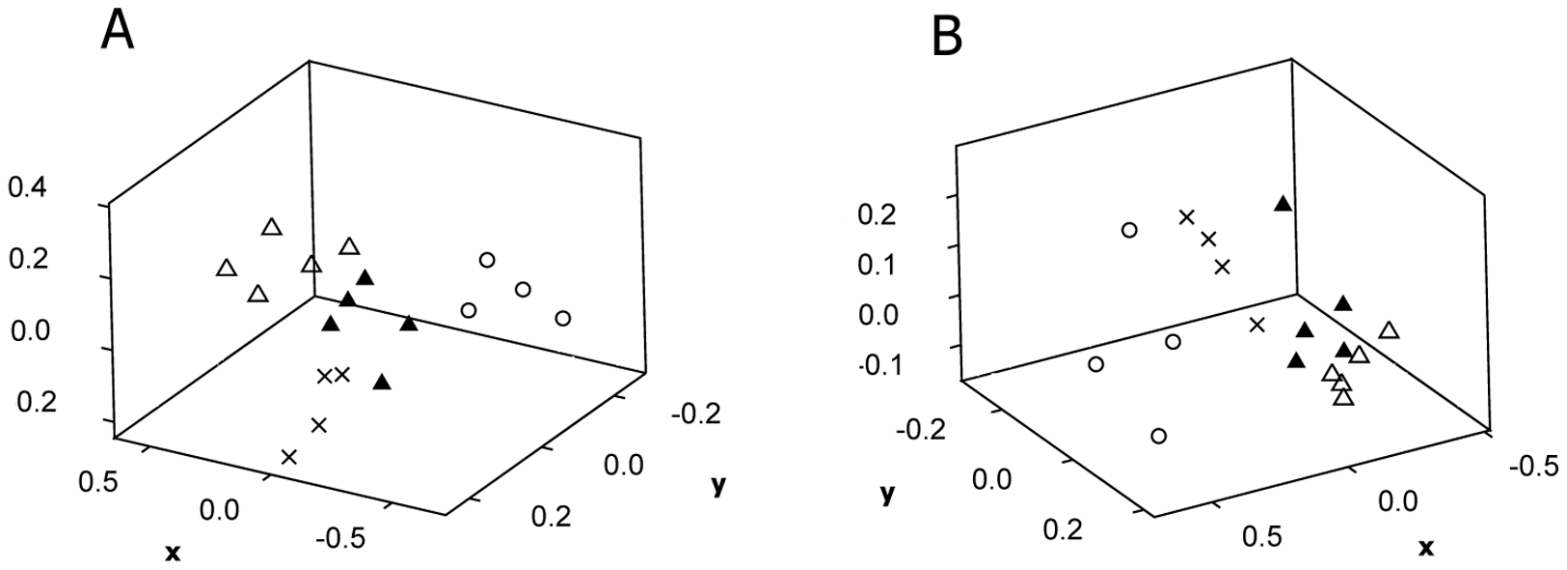
Effects of 4-n-nonylphenol on soil bacterial community structure. Effects of 4-n-nonylphenol (NP) on soil bacterial community structure, as shown from nmMDS of data from T-RFLP analyses after 28 (a) and 70 (b) days of incubation. Legends: Treatment NP low (Δ; 0.5 mg kg^−1^ dry soil) and NP high (▴; 10 mg kg^−1^ dry soil), solvent control (×; no chemical added) and original soil sample (○).

Less evident effects were observed for the single chemical treatments with 4-n-nonylphenol. Analysis of the bacterial community structure indicated an increasing change in the T-RFLP fingerprints with time, with statistically significant differences due to chemical treatment in nmMDS-analysis at 28 d (overall ANOSIM R = 0.56, p = 0.0001) and 70 d (overall ANOSIM R = 0.67, p = 0.0001) (Fig. 3, Fig. S3). A concentration effect was also evident at 28 d (R = 0.42, p = 0.035). The T-RFLP fingerprints of the fungal community upon treatment with 4-n-nonylphenol showed a similar pattern (Fig. S4), however, with larger variation between replicate samples giving lower ANOSIM R-values (range: 0.25–0.52).

**Figure 4:**
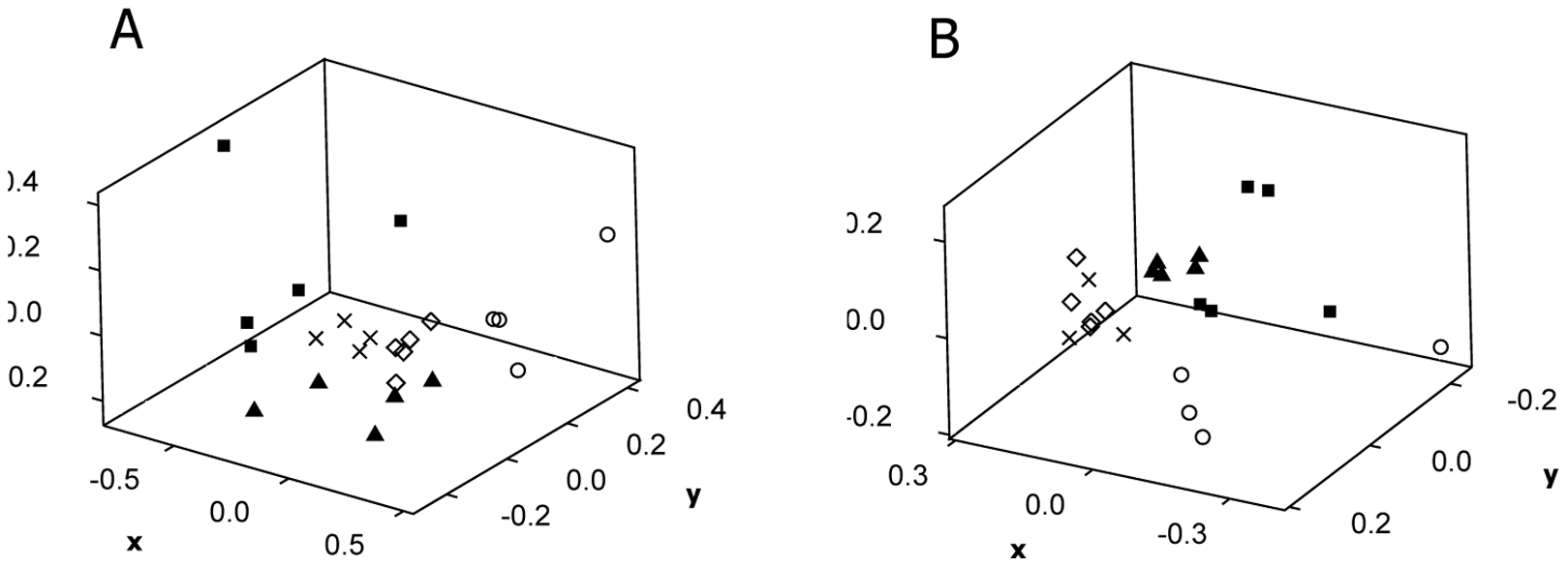
Effects of picoxystrobin and 4-n-nonylphenol on soil microbial community structure. Effects of picoxystrobin (PI) and 4-n-nonylphenol (NP) as single chemicals and in mixture on soil bacterial (a) and fungal (b) community structure, as shown from nmMDS of data from T-RFLP analyses after 28 days of incubation. Legends: Treatment PI high (▪; 10 mg kg^−1^ dry soil), NP high (▴; 10 mg kg^−1^ dry soil), Mix high (⋄; 10 mg picoxystrobin and 10 mg 4-n-nonylphenol kg^−1^ dry soil), solvent control (×; no chemical added) and original soil sample (○).

The effects of the mixture treatments (Fig. S5 and S6) could only be separated from the solvent control after 28 and 70 d for the T-RFLP fingerprints of the soil bacterial community (R = 0.73, p = 0.007 and R = 0.44, p = 0.04, respectively) and after 70 d for the soil fungal community (R = 0.72, p = 0.007). When comparing the mixture treatments with the single treatments, the more prominent alteration in T-RFLP fingerprints originated from picoxystrobin as a single chemical (10 mg kg^−1^ dry soil), with the largest separation between treatment effects observed after 28 d both for the soil bacterial (Fig. 4a) and soil fungal (Fig. 4b) community structure (overall ANOSIM R = 0.61 for both analyses). At this time-point also the single chemical treatment with 4-n-nonylphenol (10 mg kg^−1^ dry soil) could be singled out, with statistically significant changes in T-RFLP fingerprint as compared to the solvent control and other chemical treatments.

**Figure 5:**
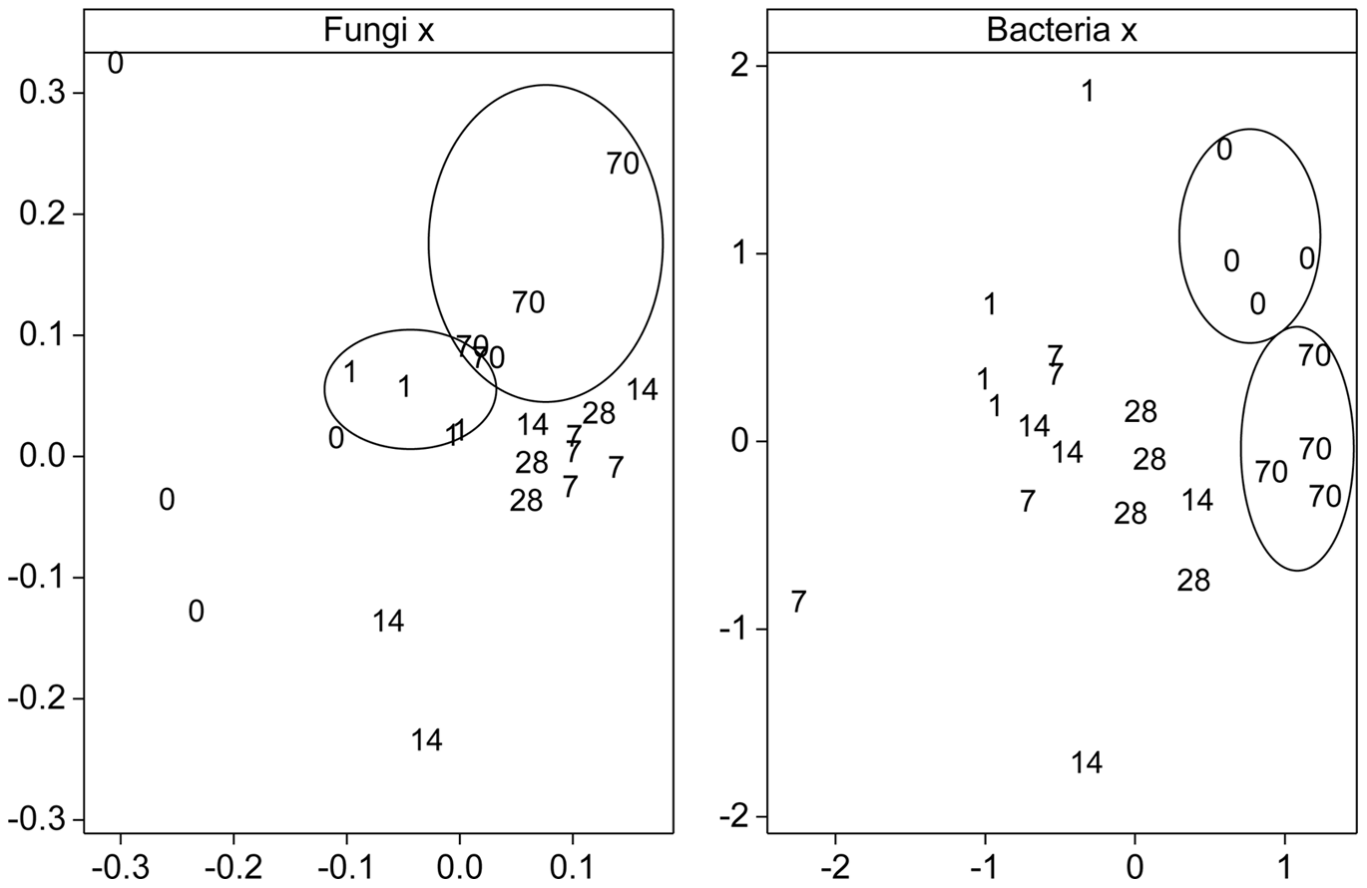
Duration of effects of chemicals on soil microbial community structure. Representative pattern (exemplified by the solvent control treatment) for observed clustering of T-RFLP fingerprints of soil microbial communities at day 0, 1, 7, 14, 28, and 70 after addition of chemicals, shown for fungi (left panel) and bacteria (right panel). Small distance between start (0) and end (70) of the incubation period indicates only minor differences in T-RFLP fingerprints, and imply transient effects of the chemicals.

The duration of the observed changes in T-RFLP fingerprints of the soil microbial communities under the different chemical treatments was examined through nmMDS analysis of the grouping of the time-points for T-RFLP analyses. In general, these results indicated that the observed changes were transient. There was a more rapid re-establishment of the original T-RFLP fingerprints in the soil bacterial (i.e. closer resemblance between time-points 0 and 70) as compared to the soil fungal community (Fig. 5).

### Degradation of Chemicals

**Figure 6:**
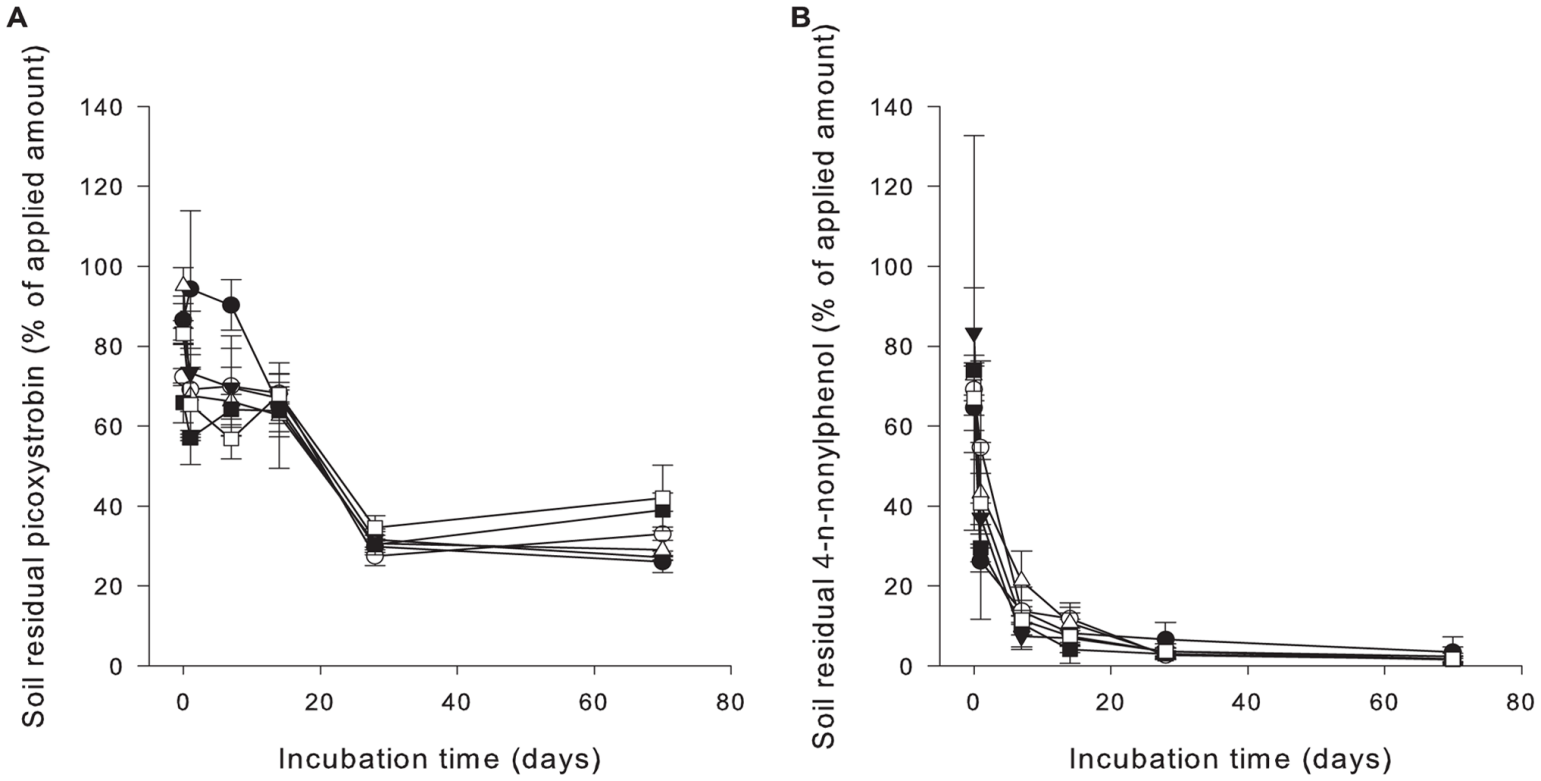
Degradation of picoxystrobin and 4-n-nonylphenol in soil. Degradation curve for picoxystrobin (a) and 4-n-nonylphenol (b) in soil, showing similar pattern and similar percentage degradation during the incubation period for all treatments with picoxystrobin and 4-n-nonylpyhenol, respectively, regardless of initial concentration or mixture situation. Mean values ± SD. Legends: Treatment PI low (a) and NP low (b) (•; PI = 2 mg and NP = 0.5 mg kg^−1^ dry soil), PI high (a) and NP high (b) (○; PI and NP = 10 mg kg^−1^ dry soil), Mix low (▾; PI = 2 mg and NP = 0.5 mg kg^−1^ dry soil), Mix NP high (▵;PI = 2 mg and NP = 10 mg kg^−1^ dry soil), Mix PI high (▪;PI = 10 mg and NP = 0.5 mg kg^−1^ dry soil), and Mix high (□; PI and NP = 10 mg kg^−1^ dry soil).

Soil chemical residue was analysed day 0, 1, 7, 14, 28 and 70 of the incubation period. A lag phase was observed before the degradation of picoxystrobin commenced (Fig. 6a), while 4-n-nonylphenol was rapidly degraded in the soil (Fig. 6b). Calculations of soil half-lives (Table 5) were based on data from both single chemicals and mixtures as the degradation patterns were similar for these. Due to very rapid initial degradation of 4-n-nonylphenol in the low concentration treatments (0.5 mg kg^−1^ dry soil), the soil half-lives were here estimated for the 0–14 days and 14–70 days separately. For the other treatments the estimates were made for 0–28 days and 28–70 days. Soil half-life for picoxystrobin for the 28–70 day period was not calculated due to a measured increase in the soil residual concentrations at the end of the incubation period, hence, not allowing a reliable estimate of the long term degradation rate.

**Table 5:** Estimated degradation half-lives (DT50) for picoxystrobin and 4-n-nonylphenol in a sandy loam soil.

Start concentrations	DT50 initial	r^2^	DT50 2^nd^ phase	r^2^
2 mg picoxystrobin kg^−1^ dry soil^a^	20.0±2.5	0.87	300±170	0.74
10 mg picoxystrobin kg^−1^ dry soil^a^	26.0±4.0	0.74		
0.5 mg 4-n-nonylphenol kg^−1^ dry soil^b^	3.0±1.0	0.85	42±2	0.58
10 mg 4-n-nonylphenol kg^−1^ dry soil^a^	6.5±0.5	0.92	52±21	0.89

a Initial phase 0–28 days, 2^nd^ phase 28–70 days,

b Initial phase 0–14 days, 2^nd^ phase 14–70 days.

Calculations are based on results from both single chemical and mixture experiments. (The test statistic of p<0.05 is valid for all estimated DT50-values.).

**Figure 7:**
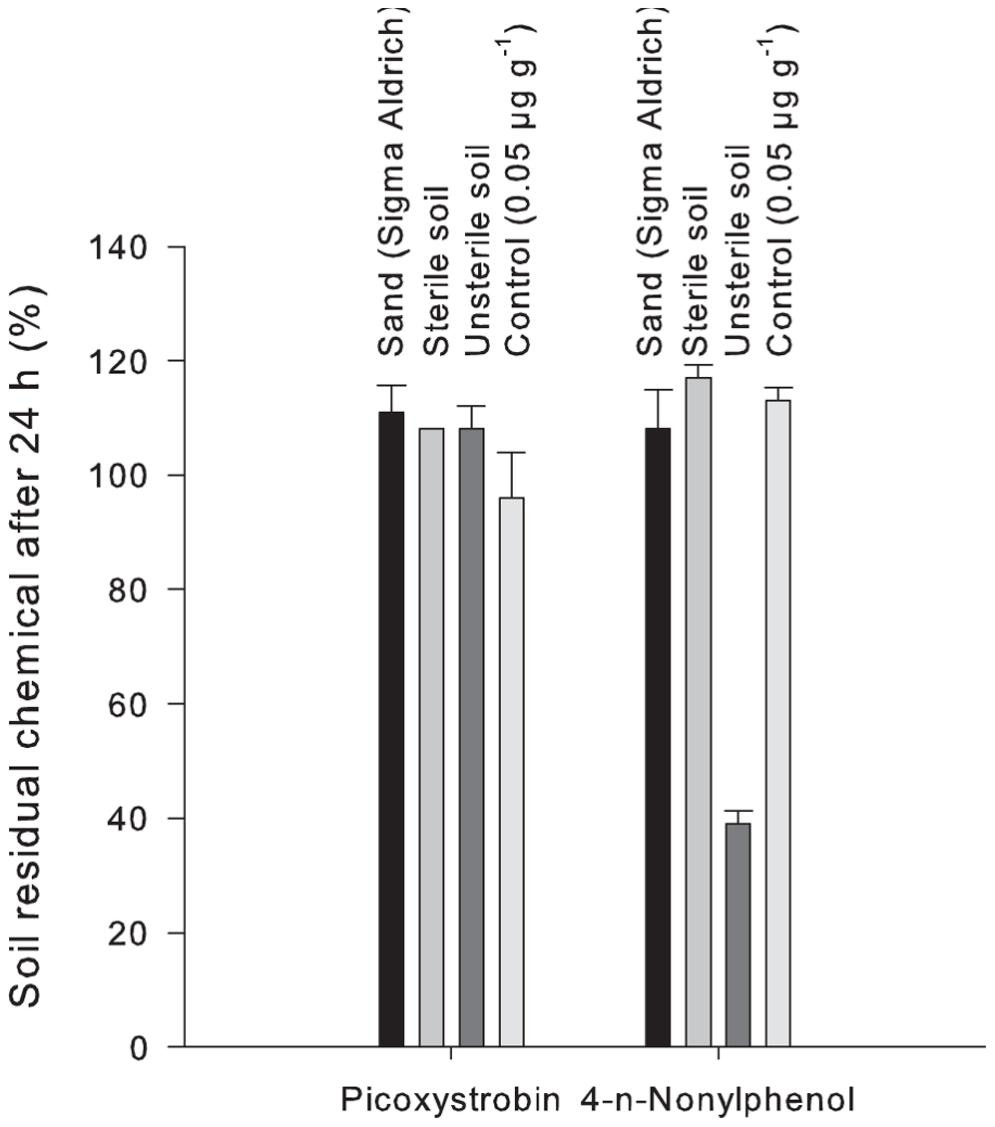
Microbial degradation of picoxystrobin and 4-n-nonylphenol in soil. Laboratory test results for degradation of picoxystrobin and 4-n-nonylphenol in selected media during 24 h, showing rapid degradation of 4-n-nonylphenol in unsterile soil.

The rapid initial degradation of 4-n-nonylphenol was confirmed and shown to be microbially mediated (Fig. 7).

## Discussion

### Significance and Duration of Observed Effects

#### Total soil microbial activity

Our findings of a negative effect of picoxystrobin on individual measurements of CO_2_ development, but not cumulative values, were in accordance with previous reports. Approval guidelines for plant protection products require documentation of effects on carbon and nitrogen mineralization, and only transient effects on nitrogen and carbon mineralization have been reported for picoxystrobin [8]. This fungicide inhibits mitochondrial respiration by blocking electron transfer at the Qo centre of cytochrome bc1 [7], and, hence, can be expected to affect the soil fungal community. We did not, however, expect an adverse effect in bacteria. The observed lag-phase before on-set of degradation of picoxystrobin (Fig. 6a) could correspond with a negative initial effect on respiration activity and microbial degradation activity. This may, however, not be concluded from the results as bacterial growth on anthropogenic substrates in soil is generally preceded by a lag-phase to allow adaptation to a new substrate. The observed slowing of the degradation rates after 28 days (Table 5) indicates limitations to microbial activity (e.g. oxygen limitation, occurrence of inhibiting factors), reduced bioavailability of the compound (e.g. time-dependent sorption) and/or a negative effect of picoxystrobin on the soil microbial community.

Our results indicated stimulating effects of high levels of 4-n-nonylphenol on soil respiration that could, in part, be explained by an increase in soil microbial activity level. As an endocrine-disrupting agent, we did not expect adverse effects of 4-n-nonylphenol on the soil bacterial community. Our findings are in accordance with others reporting low risk of adverse effects of nonylphenol in environmentally relevant concentrations on soil fungi, but with a stated need for more in-depth studies [34]. This study shows stimulation of specific fungal strains during long-term exposure, as well as appreciable sorption of nonylphenol in soil. Others have reported toxic effects of nonylphenol on soil microbes [4]. However, the general picture is that of rapid and complete mineralization in a wide range of soils [35].

#### Soil microbial community structure and degradation of chemicals

The observed changes in the T-RFLP patterns of the soil bacterial and fungal communities during the lag-phase before onset of picoxystrobin degradation (Fig. 6a), are in agreement with the general assumption of adaptation of the microbial community to a new substrate. The strobilurin mode of action is, however, typically very fast acting [6], and it may not be ruled out that the observed rapid effects on T-RFLP patterns are, in part, caused by negative effects of the fungicide. We observed a bi-phasic degradation, with initial degradation rate (DT50) in correspondence with previous reports [8]. However, in our studies the levels of picoxystrobin stabilized after 28 days, possibly due to sorption and reduced bioavailability [8], [36]. The estimated degradation half-lives (DT50) for picoxystrobin assume that the analysed fraction is the total residual fraction available for degradation. The test for initial rapid degradation (Fig. 7, left) did not indicate a rapid sorption in moist soil, but specific sorption studies were not performed. Picoxystrobin is, however, classified as only slightly mobile [36], and non-bioavailable residues are expected to be above 20% after 100 days [8]. This would explain the lack of a concentration effect beyond 28 days. Alternatively this could be due to a lasting effect on the soil microbial community, as indicated by the lasting significant differences in T-RFLP-fingerprints in picoxystrobin treated soils compared to the solvent control. Similar percentage levels of degradation of picoxystrobin were observed for the two concentrations tested, meaning that actual concentration levels remaining in the treatments with high concentrations of picoxystrobin were much higher after 28 days than for the treatments with low concentrations. There was, however, no observable concentration effect at 70 days between the T-RFLP-profiles.

This observed levelling out of the residual soil concentration of picoxystrobin after 28 days together with the lack of a concentration effect at 70 days, indicated that the observed transience of the effects on the soil microbial community was coupled to decreasing bioavailability of the chemical. Time-dependent sorption might be the reason for reduced degradation and bioavailability of the pesticide after 70 days [37]. Observed major metabolites of picoxystrobin from degradation under aerobic conditions in soil are reported to have mean half-lives in the range of 14–29 days [8]. The chemical analyses performed in our study did not include metabolites, and we may, hence, not rule out any effects of these in our short-term laboratory experiment.

Although 4-n-nonylphenol was found to stimulate soil respiration, the effects on the microbial community structure were less evident. The observed changes in the T-RFLP fingerprints for the soil bacterial community developed more slowly after 4-n-nonylphenol treatment than for picoxystrobin. This indicated a change induced by microbial growth on the chemical, in accordance with the rapid disappearance of 4-n-nonylphenol from the soil through microbial degradation (Fig. 7, right). Residual amounts approached zero both when measured in percentage and in actual amounts. Nonylphenol originates from anthropogenic activity and accumulates and persists in environmental compartments characterized by high organic content, such as sewage sludge [3]. We observed rapid degradation in agricultural soil of comparatively low organic carbon content (quite on the average for large parts of the Norwegian agricultural area). This rapid degradation in an aerobic soil environment is in accordance with previous studies [35], [38]. There are, however, reports of field studies with fast initial degradation, while some residues might persist [4]. The occurrence of metabolites of 4-n-nonylphenol was not measured in this study, but the observed stimulation of respiration activity by 4-n-nonylphenol addition as compared to the solvent control, was not large enough to account for the observed disappearance of the chemical through the incubation period. This indicated that the chemical was not completely mineralized. Results from a mineralization study with a range of soils show the conversion of around 40% of applied nonylphenol to CO_2_ during a 40 day incubation period [39].

Our results indicated shorter duration of the effects of the chemicals in the soil bacterial as compared to the soil fungal community. This was expected due to shorter generation times enabling a faster re-establishment of the community structure. Overall, our results were in accordance with this, showing smaller differences in T-RFLP fingerprints between start and end of the experiment for the former (Fig. 5). The more statistically significant effects of the treatments were however shown for bacteria, while the changes in the soil fungal community were less consistent and varied a lot between replicate samples.

### Implications of a Mixture Situation on the Effects of Picoxystrobin on the Soil Microbial Community

Analyses of the effects of mixture treatments on the soil microbial community structure indicated that the two chemicals might have antagonistic effects. Established concepts to estimate biological effects of chemical mixtures rely on data available for single chemicals [40], [41]. Single chemicals vary in how they excerpt their effect and the effects of a mixture cannot be found directly from the different constituents’ independent effects. One can observe a wide variety of effects due to synergism, antagonism or other forms of interactions, and the common concepts of concentration addition and independent action often come short. Despite the expected and indicated differences in modes of action of the two chemicals, we observed less evident changes in T-RFLP patterns in soils treated with the chemicals in mixture, as compared to the single chemical treatments. Our results showed that statistically significant different T-RFLP patterns, for both the soil bacterial and fungal communities, resulted from treatments with picoxystrobin, 4-n-nonylphenol, and a mixture of these. However, the changes in T-RFLP patterns evolved more slowly and were less pronounced for the mixture than for the single chemical treatments, as compared to the solvent control. Further, the negative effects of low levels of picoxystrobin on respiration were apparently remediated when mixing with high concentration of 4-n-nonylphenol (Mix NP high), while high levels of picoxystrobin nulled the positive effects of high levels of 4-n-nonylphenol (Mix high). In a practical mixture situation in the field, the expected environmental concentrations of nonylphenol can from this not be expected to increase the potentially adverse effects of picoxystrobin.

To assure the validity in extrapolation of the observed effects to a risk assessment situation in the field, the results need to be verified in field/semi-field/mesocosm trials. These lab studies do not take into account the additional stress the soil microbial community experience in nature from climatic conditions (drought, frost), predators, competition, or food shortage, nor the effects of alteration in the soil microbial community structure on other parts of the soil fauna. The studied mixture is relevant in an agricultural context and the results should be possible to extrapolate to a certain degree to other nonylphenols and strobilurin fungicides. However, care should be taken to consider the variety of physico-chemical and toxicological properties of the different strobilurins [6] as well as the differences in estrogenicity, sorption properties and degradation rates of different isomers of linear and branched nonylphenols [42]. Further, due to the general importance of soil properties for the sorption of chemicals, extrapolation of results from this sandy loam soil with low organic carbon content should be done with care.

### Methodological Considerations

Our results showed that both soil microbial respiration activity and community structure was affected by the chemicals. They also illustrate the need for the use of several measures to be able to assess effects of chemicals in soil with a minimum degree of certainty. We found statistically significant negative effects of picoxystrobin on activity levels as shown by CO_2_ development, but no statistically significant differences on cumulative CO_2_-values (Table 4) due to too large variability between replicate samples. This was despite observable trends in the data (Fig. 1). Further, the T-RFLP results indicated a need for frequent sampling shortly after addition of chemicals, and possibly the resolution of the sampling was too low to capture the effects on the soil fungal community structure as the largest spread in these data as shown by nmMDS was found after 7 days (Fig. 2). The community fingerprints arising from T-RFLP analyses depend on the PCR-primers and restriction enzymes utilized, and cannot easily be interpreted in terms of ecological relevance of the observed effects. This was ameliorated through correspondence with effects on soil respiration activity. Our studies showed that the T-RFLP technique could be used as a valuable tool in elucidating how rapid and to what extent effects (positive or negative) of the chemicals in the soil microbial community can be expected.

Our results for the fungicide picoxystrobin show that the mere measurement of changes in soil chemical concentrations for this moderately sorbing chemical could not be used as an indication of when to expect effects, while exposure assessment in ecological risk assessment is often restricted to external exposure like concentrations in water, soil or sediment. However it has been recognized that in natural ecosystems, in particular in soils and sediments, the amount of chemicals truly available for uptake into organisms is frequently only a fraction of the total amount present, due to a complex environmental behaviour [43]. Measurements of residual chemical concentrations in our test system was, however, shown to be a valuable tool for explaining the observed changes in soil microbial community structure and respiration activity and elucidating the concept of a chemicals bioavailability, and should be included in effects assessments.

### Conclusions

In summary, we have studied the degradation and effects of picoxystrobin and 4-n-nonylphenol on the soil microbial community structure and respiration activity in a sandy loam soil. The fungicide picoxystrobin was shown to decrease the soil microbial activity, while 4-n-nonylphenol caused an increase. These effects were accompanied by statistically significant changes in the T-RFLP fingerprints for the soil microbial community that were still detectable, but small, after 70 days. A mixture situation relevant for assessing the environmental risk of fungicide application in years with sewage sludge amendment of agricultural soil was tested. These results implied that a mixture of picoxystrobin and 4-n-nonylphenol will not have more adverse effects on soil respiration or the soil microbial community structure than the single chemicals. They indicated that the chemicals affected different parts of the soil microbial community, and resulted in a low net effect of the chemicals in mixture. The presented results do, however, not allow us to propose an explanation for the mechanisms causing this response.

In conclusion, our results imply that the application of picoxystrobin and nonylphenol to agricultural soil in environmentally relevant and worst-case concentrations will not cause irreversible effects on soil microbial respiration and community structure. Further, a mixture situation with the fungicide picoxystrobin and nonylphenol from sewage sludge application, will not increase any potentially adverse effects of picoxystrobin on the soil microbial community. In a wider perspective, these results illustrate that there is a need and possibility to refine today’s risk assessment procedures to encompass the study of both soil bacterial and fungal communities, both broad-scale measures and genomics, and interpret the results in relation to residual soil chemical concentrations.

## Supporting Information

Figure S1 **Effects of picoxystrobin on soil bacterial community structure.** Effects of picoxystrobin (PI) on soil bacterial community structure, as shown from nmMDS of data from T-RFLP analyses. Legends: Treatment PI low (□; 2 mg kg^−1^ dry soil) and PI high (▪; 10 mg kg^−1^ dry soil), solvent control (X; no chemical added) and original sample (○).
(TIF)

Figure S2 **Effects of picoxystrobin on soil fungal community structure.** Effects of picoxystrobin (PI) on soil fungal community structure, as shown from nmMDS of data from T-RFLP analyses. Legends: Treatment PI low (□; 2 mg kg^−1^ dry soil) and PI high (▪; 10 mg kg^−1^ dry soil), solvent control (X; no chemical added) and original sample (○).
(TIF)

Figure S3 **Effects of 4-n-nonylphenol on soil bacterial community structure.** Effects of 4-n-nonylphenol (NP) on soil bacterial community structure, as shown from nmMDS of data from T-RFLP analyses. Legends: Treatment NP low (Δ; 0.5 mg kg^−1^ dry soil) and NP high (▴; 10 mg kg^−1^ dry soil), solvent control (X; no chemical added) and original sample (○).
(TIF)

Figure S4 **Effects of 4-n-nonylphenol on soil fungal community structure.** Effects of 4-n-nonylphenol (NP) on soil fungal community structure, as shown from nmMDS of data from T-RFLP analyses. Legends: Treatment NP low (Δ; 0.5 mg kg^−1^ dry soil) and NP high (▴; 10 mg kg^−1^ dry soil), solvent control (X; no chemical added) and original sample (○).
(TIF)

Figure S5 **Effects of picoxystrobin and 4-n-nonylpyhenol on soil bacterial community structure.** Effects of picoxystrobin (PI) and 4-n-nonylphenol (NP) on soil bacterial community structure, as shown from nmMDS of data from T-RFLP analyses. Legends: Treatment PI high (▪; 10 mg kg^−1^ dry soil), NP high (▴; 10 mg kg^−1^ dry soil), Mix high (⋄; 10 mg picoxystrobin and 4-n-nonylphenol kg^−1^ dry soil), solvent control (X; no chemical added) and original sample (○).
(TIF)

Figure S6 **Effects of picoxystrobin and 4-n-nonylpyhenol on soil fungal community structure.** Effects of picoxystrobin (PI) and 4-n-nonylphenol (NP) on soil fungal community structure, as shown from nmMDS of data from T-RFLP analyses. Legends: Treatment PI high (▪; 10 mg kg^−1^ dry soil), NP high (▴; 10 mg kg^−1^ dry soil), Mix high (⋄; 10 mg picoxystrobin and 4-n-nonylphenol kg^−1^ dry soil), solvent control (X; no chemical added) and original sample (○).
(TIF)

Table S1 **Summary of results from nmMDS (STRESS; k = 3 dimensions, Bray-Curtis distance measure) and ANOSIM (overall R) of T-RFLP fingerprints for the soil microbial community after different chemical treatments.**
(PDF)
