# Supplementary figures and images for: Effects of Picoxystrobin and 4-n-Nonylphenol on Soil Microbial Community Structure and Respiration Activity
Source: PLoS One. 2013 Jun 20;8(6):e66989. doi: 10.1371/journal.pone.0066989 (PMC3688581; doi:10.1371/journal.pone.0066989)

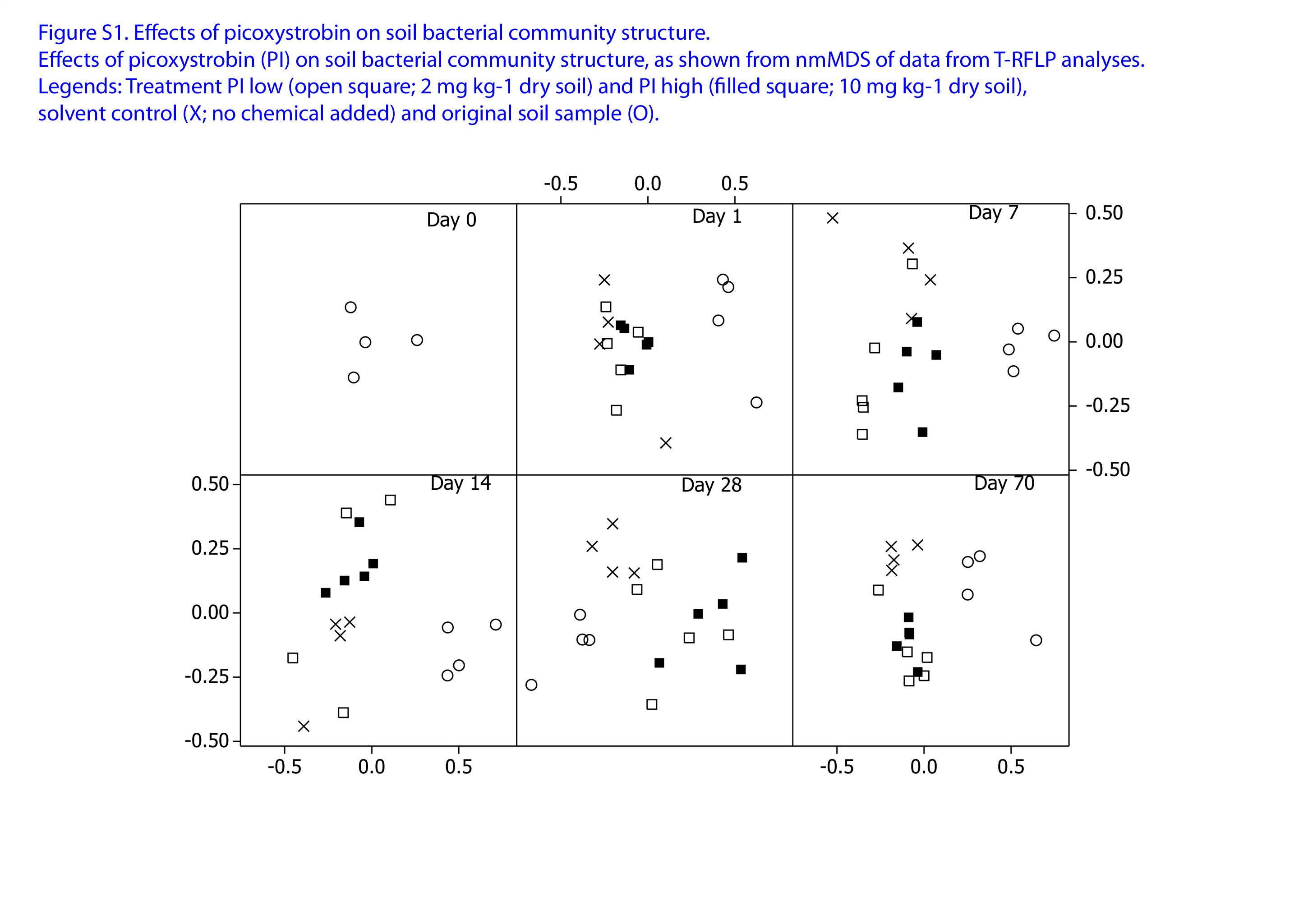

Supplement: Figure S1 — Effects of picoxystrobin on soil bacterial community structure. Effects of picoxystrobin (PI) on soil bacterial community structure, as shown from nmMDS of data from T-RFLP analyses. Legends: Treatment PI low (□; 2 mg kg−1 dry soil) and PI high (▪; 10 mg kg−1 dry soil), solvent control (X; no chemical added) and original sample (○). (TIF) [file pone.0066989.s001.tif]

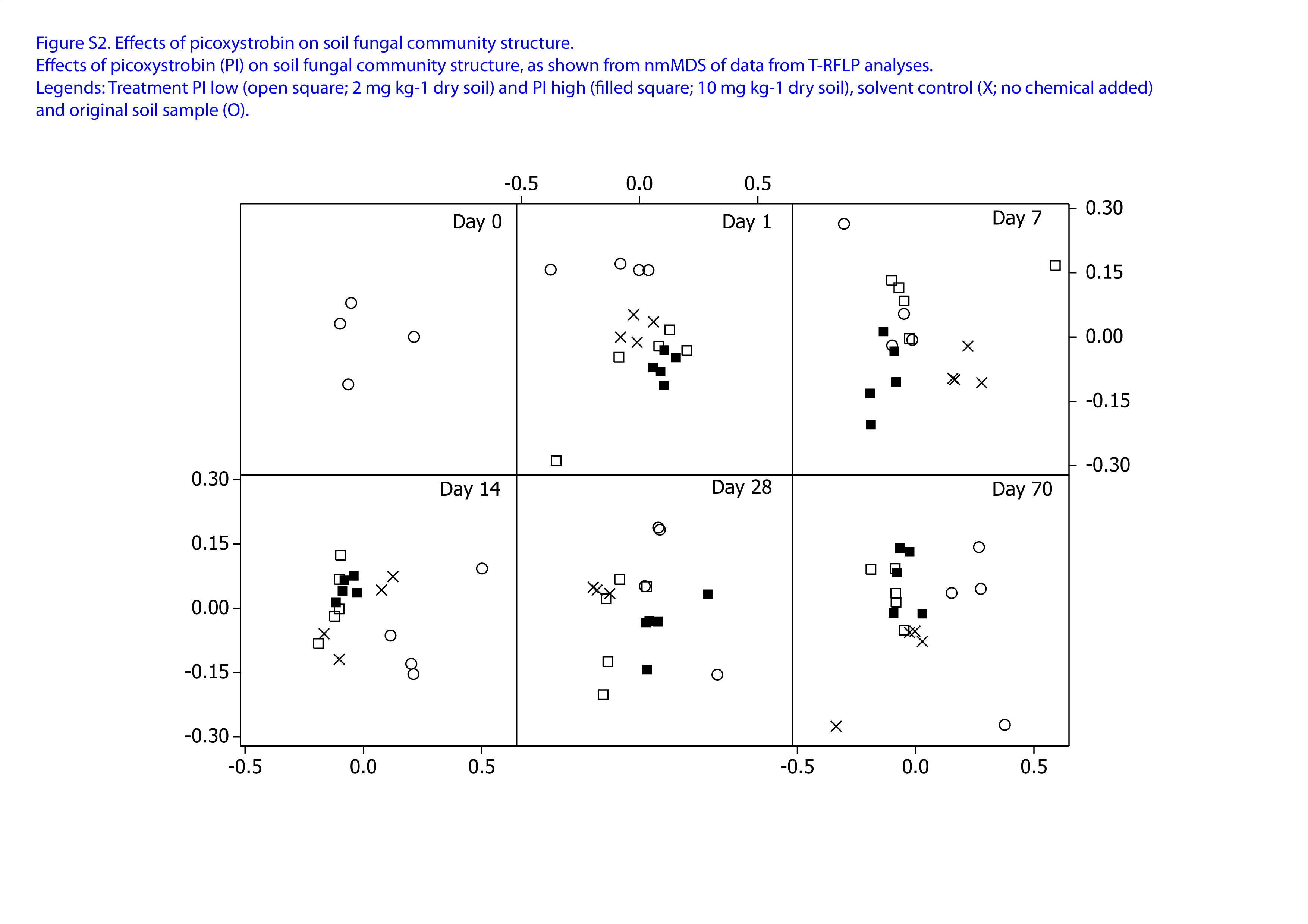

Supplement: Figure S2 — Effects of picoxystrobin on soil fungal community structure. Effects of picoxystrobin (PI) on soil fungal community structure, as shown from nmMDS of data from T-RFLP analyses. Legends: Treatment PI low (□; 2 mg kg−1 dry soil) and PI high (▪; 10 mg kg−1 dry soil), solvent control (X; no chemical added) and original sample (○). (TIF) [file pone.0066989.s002.tif]

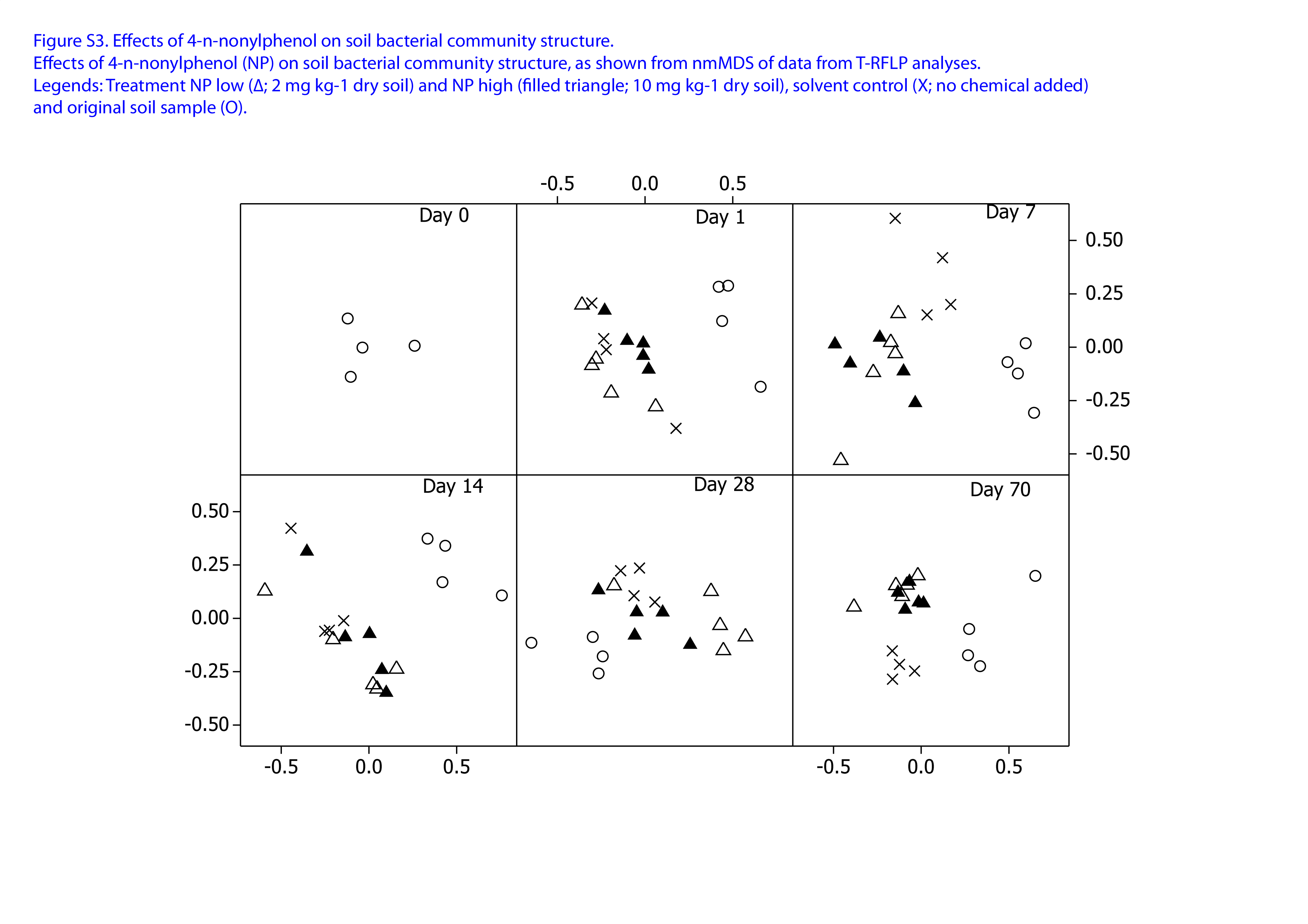

Supplement: Figure S3 — Effects of 4-n-nonylphenol on soil bacterial community structure. Effects of 4-n-nonylphenol (NP) on soil bacterial community structure, as shown from nmMDS of data from T-RFLP analyses. Legends: Treatment NP low (Δ; 0.5 mg kg−1 dry soil) and NP high (▴; 10 mg kg−1 dry soil), solvent control (X; no chemical added) and original sample (○). (TIF) [file pone.0066989.s003.tif]

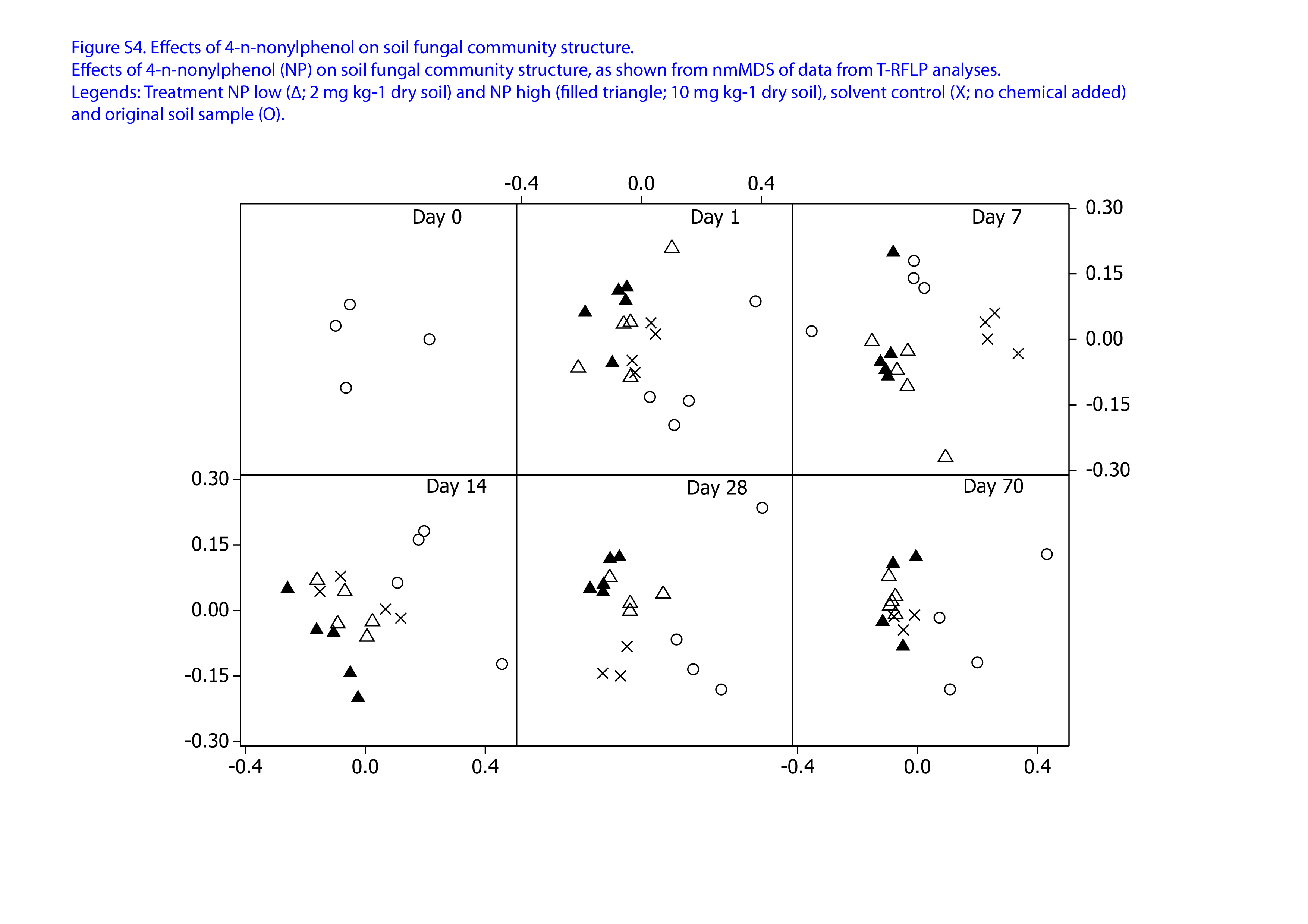

Supplement: Figure S4 — Effects of 4-n-nonylphenol on soil fungal community structure. Effects of 4-n-nonylphenol (NP) on soil fungal community structure, as shown from nmMDS of data from T-RFLP analyses. Legends: Treatment NP low (Δ; 0.5 mg kg−1 dry soil) and NP high (▴; 10 mg kg−1 dry soil), solvent control (X; no chemical added) and original sample (○). (TIF) [file pone.0066989.s004.tif]

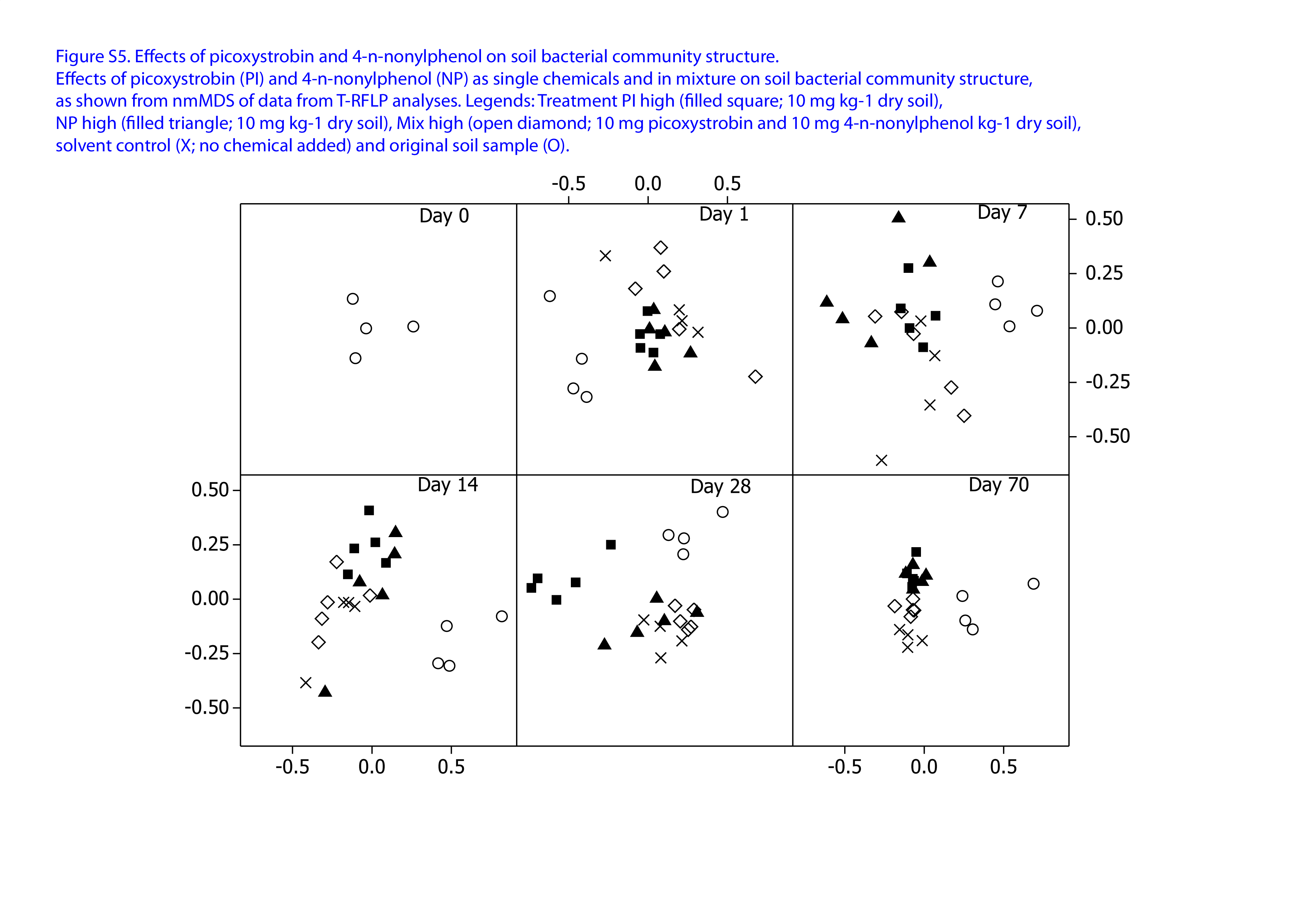

Supplement: Figure S5 — Effects of picoxystrobin and 4-n-nonylpyhenol on soil bacterial community structure. Effects of picoxystrobin (PI) and 4-n-nonylphenol (NP) on soil bacterial community structure, as shown from nmMDS of data from T-RFLP analyses. Legends: Treatment PI high (▪; 10 mg kg−1 dry soil), NP high (▴; 10 mg kg−1 dry soil), Mix high (⋄; 10 mg picoxystrobin and 4-n-nonylphenol kg−1 dry soil), solvent control (X; no chemical added) and original sample (○). (TIF) [file pone.0066989.s005.tif]

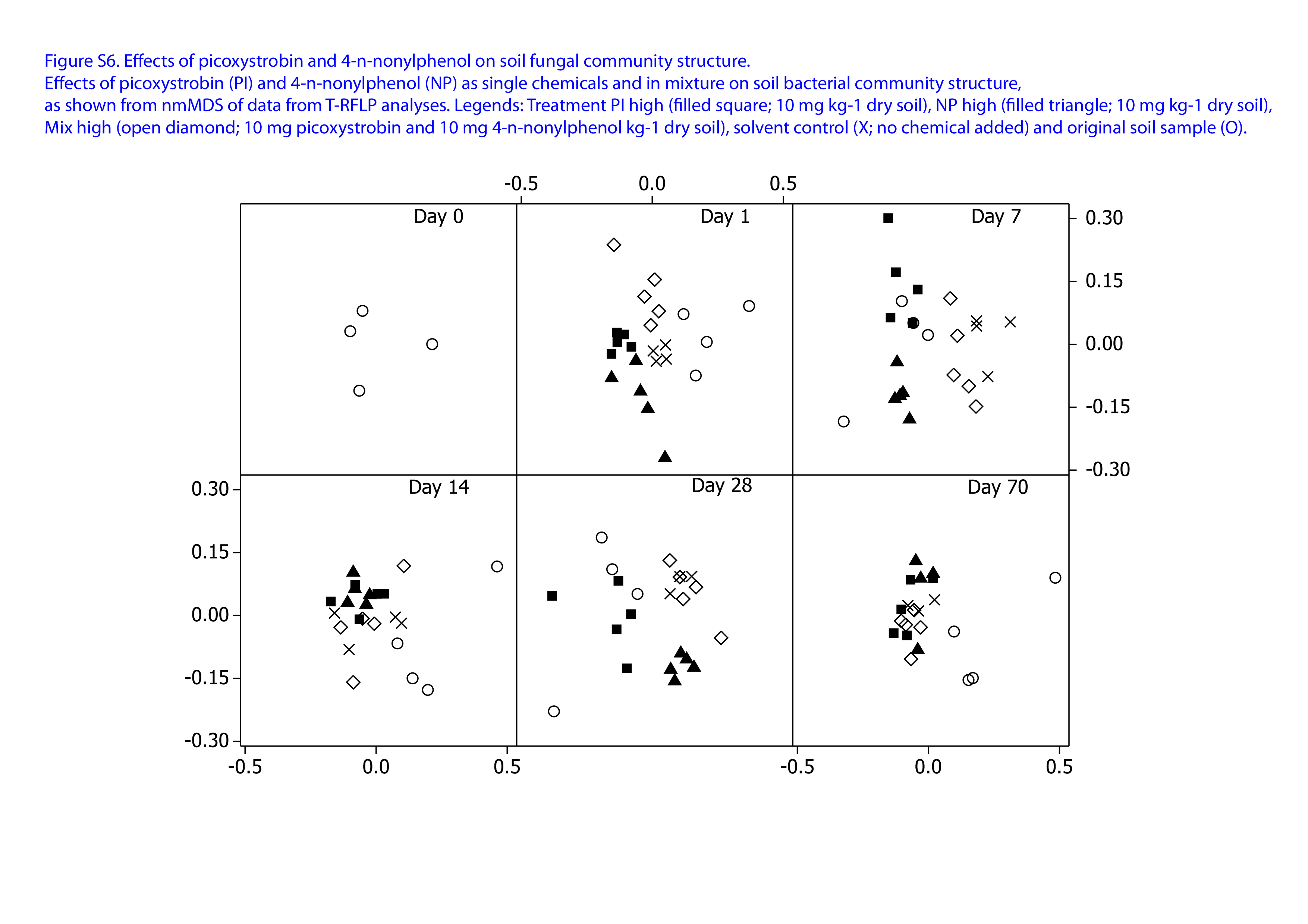

Supplement: Figure S6 — Effects of picoxystrobin and 4-n-nonylpyhenol on soil fungal community structure. Effects of picoxystrobin (PI) and 4-n-nonylphenol (NP) on soil fungal community structure, as shown from nmMDS of data from T-RFLP analyses. Legends: Treatment PI high (▪; 10 mg kg−1 dry soil), NP high (▴; 10 mg kg−1 dry soil), Mix high (⋄; 10 mg picoxystrobin and 4-n-nonylphenol kg−1 dry soil), solvent control (X; no chemical added) and original sample (○). (TIF) [file pone.0066989.s006.tif]
