# Supplementary material for: Effects of Picoxystrobin and 4-n-Nonylphenol on Soil Microbial Community Structure and Respiration Activity
Source: PLoS One. 2013 Jun 20;8(6):e66989. doi: 10.1371/journal.pone.0066989 (PMC3688581; doi:10.1371/journal.pone.0066989)
Supplement: Table S1 — Summary of results from nmMDS (STRESS; k = 3 dimensions, Bray-Curtis distance measure) and ANOSIM (overall R) of T-RFLP fingerprints for the soil microbial community after different chemical treatments. (PDF) [file pone.0066989.s007.pdf]

Table S1. Summary of results from nmMDS (STRESS; k=3 dimensions, Bray-Curtis distance measure) and ANOSIM (overall R) of T-RFLP fingerprints for the soil microbial community after different chemical treatments.

| | Factors | Time (d) | STRESS | R* |
|---|---|---|---|---|
| Bacteria | Picoxystrobin[1] | 1 | 0.07 | 0.52 |
| | | 7 | 0.06 | 0.65 |
| | | 14 | 0.07 | 0.59 |
| | | 28 | 0.05 | 0.63 |
| | | 70 | 0.05 | 0.65 |
| | 4-n-Nonylphenol[2] | 1 | 0.07 | 0.50 |
| | | 7 | 0.06 | 0.56 |
| | | 14 | 0.05 | 0.44 |
| | | 28 | 0.06 | 0.56 |
| | | 70 | 0.04 | 0.67 |
| | Mixture[3] | 1 | 0.09 | 0.41 |
| | | 7 | 0.10 | 0.50 |
| | | 14 | 0.06 | 0.52 |
| | | 28 | 0.07 | 0.61 |
| | | 70 | 0.08 | 0.62 |
| Fungi | Picoxystrobin[1] | 1 | 0.06 | 0.41 |
| | | 7 | 0.06 | 0.47 |
| | | 14 | 0.05 | 0.49 |
| | | 28 | 0.06 | 0.37 |
| | | 70 | 0.07 | 0.39 |
| | 4-n-Nonylphenol[2] | 1 | 0.09 | 0.25 |
| | | 7 | 0.08 | 0.52 |
| | | 14 | 0.06 | 0.41 |
| | | 28 | 0.04 | 0.49 |
| | | 70 | 0.06 | 0.44 |
| | Mixture[3] | 1 | 0.10 | 0.44 |
| | | 7 | 0.08 | 0.61 |
| | | 14 | 0.08 | 0.38 |
| | | 28 | 0.07 | 0.61 |
| | | 70 | 0.09 | 0.48 |

*Significance level $p<0.001$. [1]: PI low, PI high, solvent control, original soil. [2]: NP low, NP high, solvent control, original soil. [3]: PI high, NP high, Mixture high, solvent control, original soil.
